# RNA function and phosphorus use by photosynthetic organisms

**DOI:** 10.3389/fpls.2013.00536

**Published:** 2013-12-26

**Authors:** John A. Raven

**Affiliations:** ^1^Division of Plant Science, University of Dundee at the James Hutton InstituteDundee, UK; ^2^School of Plant Biology, University of Western AustraliaPerth, WA, Australia

**Keywords:** allocation, diel, growth, peptide elongation, protein synthesis

## Abstract

Phosphorus (P) in RNA accounts for half or more of the total non-storage P in oxygenic photolithotrophs grown in either P-replete or P-limiting growth conditions. Since many natural environments are P-limited for photosynthetic primary productivity, and peak phosphorus fertilizer production is inevitable, the paper analyses what economies in P allocation to RNA could, in principle, increase P-use efficiency of growth (rate of dry matter production per unit organism P). The possibilities of decreasing P allocation to RNA without decreasing growth rate include (1) more widespread down-regulation of RNA production in P-limited organisms, (2) optimal allocation of P to RNA, both spatially among cell compartments and organs, and temporally depending on the stage of growth, and (3) a constant rate of protein synthesis through the diel cycle. Acting on these suggestions would, however, be technically demanding.

## Introduction

RNA is an essential component of all living organisms, and the phosphorus (P) it contains cannot be substituted by any other element. The core role of RNA is in protein synthesis, involving mRNA, rRNA, and tRNA, but it also has (presumably) derived functions in regulation of gene expression. RNA-P is often the major non-storage form of P in photosynthetic organisms, and any attempts to increase the P-use efficiency of growth of algae and plants must include a consideration of economizing on the use of RNA. The paper first deals with the general background of the molecular biology, biochemistry, physiology and biogeochemistry, including optimal allocation of P within the organism in the context of the Growth Rate Hypothesis (Sterner and Elser, 2002). It then considers the various ways of expressing the RNA content of photosynthetic organisms, and the upper limit of the *in vivo* peptide elongation rate per ribosome. Optimal allocation and the Growth Rate Hypothesis are then revisited in the context of variations in growth rate in relation to RNA content, including temporal and spatial variations in net protein synthesis rate per unit RNA, and then the significance of protein turnover in the total rate of protein synthesis per unit RNA. The paper concludes with consideration of how plant P-use efficiency could be increased by maximizing protein synthesis rate per unit RNA at all times and in all parts of the organism.

## Background

Phosphorus (P) is an essential element for all of the organisms that have been examined (Erb et al., 2012; Reaves et al., 2012). The P in RNA in photosynthetic (and other) organisms is a major component of non-storage P. RNA has 0.091 g P per g dry matter (Table 1). Corresponding values for other P-containing organic compounds are 0.095 for DNA, 0.041 for phospholipids, 0.12–0.23 for low-molecular-mass water-soluble phosphate esters and 0.32 for inorganic phosphate (Table 1). The generally greater mass of RNA than of the sum of the other organic phosphates means that RNA accounts for half (or even more) of non-storage P in cyanobacteria, algae, and plants (reviewed by Raven, 2008, 2012, 2013c; Veneklaas et al., 2012). Occasionally, under severe P limitation permitting only very low growth, and hence protein synthesis, rates, DNA is the major P-containing component of organisms (Bertilsson et al., 2003).

**Table 1:** **Fraction of phosphorus in phosphorus-containing compounds in cells**.

**Phosphorus-containing compound**	**g phosphorus per g dry compounds**
RNA	0.091
DNA	0.095
Phospholipid	0.041
Hexose-1-phosphate or hexose-6-phosphate	0.12
Fructose-1,6-bisphosphate	0.18
ATP	0.18
Ribulose-1,5-bisphosphate	0.20
2-phosphoglycolate	0.22
Glycerate-1,3-bisphosphate	0.23
Inorganic phosphate	0.32

*From Geider and La Roche (2002), with additional calculations on low-molecular-mass water-soluble phosphate esters and on inorganic phosphate*.

The best-understood roles for RNA are in protein synthesis, involving three kinds of RNA. rRNA comprises about half of the mass of 80S ribosomes (eukaryotic cytosol) and two-thirds of the mass of 70S ribosomes (Archaea, Bacteria, mitochondria, chloroplasts), the rest being protein. Ribosomes catalyze the translation of mRNA via the addition of amino-acyl units from amino-acyl tRNA to growing polypeptide chains. mRNA is the intermediary in converting the DNA nucleotide sequence in genes into the amino acid sequence in proteins. Intron removal from mRNA transcripts by splicing is an essential part of translation, and alternative splicing means that two or more different functional proteins can be produced from a single gene (Syed et al., 2012). tRNA acts as the “adaptor” with a sequence recognizing an anticodon in mRNA corresponding to the appropriate amino acid and also a site recognizing and binding that amino acid as amino-acyl tRNA. Non-ribosomal peptide bond formation also occurs (Mocibob et al., 2010; Roy and Ibba, 2010), e.g., in the synthesis of glutathione and its polymers, phytochelatin and metallothioneins (Clemens and Peršon, 2009), and in cyclic peptide toxins of cyanobacteria (Pearson et al., 2010). However, the enzymes involved in these syntheses are themselves synthesized using rRNA, mRNA, and tRNA. There are also a number of small informational RNAs (miRNAs and siRNAs) involved in gene silencing in both unicellular and multicellular eukaryotes (Baulcombe, 2004; Molnár et al., 2007; Clemens and Peršon, 2009; Zhang et al., 2009; Kragler, 2010; Huang et al., 2011; Norden-Krichmar et al., 2011).

For the three major RNA forms, it is known that rRNA, mRNA, and tRNA function in protein synthesis in the cytosol, chloroplasts and mitochondria, with 70S ribosomes in the organelles and 80S ribosomes in the cytosol. The different location for the major RNA forms is one aspect of differences in the location of ribosomes and the site of functioning of the proteins that the ribosomes produce. The DNAs of mitochondria and chloroplasts only encode about 10% of the proteins required for functioning of the organelles; the remaining 90% or so of the proteins in the organelles are encoded in the nucleus, translated on 80S ribosomes in the cytosol, and imported into the organelles. This is taken up below in the context of the allocation of RNA between cytosol and chloroplasts and the rates of protein synthesis in those compartments. Analogous import procedures occur in the components of the endomembrane system whose lumen lacks DNA and RNA. Ribosomes, and nuclei, are absent from functional sieve tube elements; despite this, some sieve tubes are very long-lived (Raven, 1991; van Dongen et al., 2003; Kragler, 2010), and protein degradation and synthesis occur in functional sieve-tube (Fisher et al., 1992). The proteins are transcribed and translated in companion cells and move to the sieve tubes through specialized plasmodemata (van Bel and Knoblauch, 2000; van Bel, 2003). The phloem is clearly a conduit for miRNA movement from shoot to root, used in signaling related to the regulation of nutrient element content in different plant parts (Zehr, 2013).

The distribution of total RNA among rRNA, mRNA, tRNA, miRNA, and siRNA in photosynthetic organisms is not well-understood. In the ascomycotan *Saccharomyces* the ratio of tRNA to rRNA by mass is 0.11 at the highest growth rate and 0.14 at the lowest growth rate (Waldron and LaCroute, 1975). A similar ratio of tRNA to rRNA is found in *Neurospora*, also an ascomycotan (Alberghina et al., 1975). mRNA may be as little as 1% of total RNA in *Lemna* (Tobin and Klien, 1975), but can be 5% of total RNA in *Saccharomyces* (Warner, 1999). The fraction of total RNA represented by the three different kinds of RNA involved in the catalysis of protein synthesis is, then, in descending order, rRNA, tRNA, mRNA.

Turning from the molecular, cellular, and organismal to the planetary scale, P is probably the major resource limiting primary productivity on Earth over geological time periods; P is also an essential agricultural fertilizer whose economic extraction rate is predicted to decline after the 2030s, with “peak phosphorus” predicted for, 2035 (Cordell et al., 2009; Cordell and White, 2011; Raven, 2012, 2013c; Veneklaas et al., 2012), although other analyses suggest much later “peak phosphorus” (Fixen and Johnston, 2011; Scholz and Wellmer, 2013). These biogeochemical considerations, together with the abovementioned predominance of RNA-P as the major component of non-storage P in cyanobacteria, algae, and plants, are the rationale for focussing much of the rest of this article on the determinants of the effectiveness with which RNA-P is used in protein synthesis in growth and maintenance of photosynthetic organisms. By effectiveness is meant the extent to which the maximum achievable rate of protein synthesis per unit of RNA observed *in vivo* is achieved in particular photosynthetic organisms. As well as such calculations, other determinants of the effectiveness of RNA use are considered. The underlying hypothesis in such investigations is optimal allocation (Rosen, 1967), i.e., the allocation of RNA to rRNA, tRNA, and mRNA within a compartment, of RNA among compartments in a cell, and of RNA among cell types and organs, such that growth rate and, more generally, evolutionary fitness is maximized. In macroalgae and embryophytic plants with meristematic zones (net protein synthesis and protein turnover) and mature zones little or no net protein synthesis, but protein turnover, the correlation between growth and RNA content is likely to be closer in meristems than in mature structures, such as the mature leaves that are sometimes examined (Raven, 1989, 1994, 2011, 2012, 2013c; Lambers et al., 2010; Veneklaas et al., 2012). This differs from micro-organisms (such as microalgae) and in young stages of metazoans (Sterner and Elser, 2002; Elser et al., 2003).

P allocation among the kinds of RNA and their location should be considered over time to determine if there are variations in the approach to optimality over time in a diel cycle or in the life of an organ. A major preoccupation of research on the RNA content of organisms is its relation to genotypically or phenotypically determined growth rates, related to the Growth Rate Hypothesis (Sterner and Elser, 2002). The Growth Rate Hypothesis is based on the observed correlation in a number of mainly non-photosynthetic organisms of an increasing growth rate with an increasing fraction of the biomass occupied by RNA and an increasing RNA:protein ratio (Sterner and Elser, 2002; Elser et al., 2003). Furthermore, because RNA is a major component of organism P just as protein is the major component of organism N, increasing growth rate in these organisms is correlated with a decreasing N:P ratio (Sterner and Elser, 2002; Elser et al., 2003). These correlations have been related to the role of P as the ultimate resource limiting productivity on Earth (Sterner and Elser, 2002; Raven, 2012, 2013c; Veneklaas et al., 2012).

Prediction that the peak extraction rate of P fertilizer from rock phosphate will occur within the next few decades (Cordell et al., 2009; Cordell and White, 2011) has catalyzed analyses of how plant P-use efficiency (dry matter per unit plant P or, preferably in the general case, rate of dry matter production per unit plant P) can be increased (Raven, 2012, 2013c; Veneklaas et al., 2012). Such studies of P use efficiency are still important even if substantially longer times to peak P extraction (Fixen and Johnston, 2011; Scholz and Wellmer, 2013) are accepted Since RNA is usually the major component of non-storage P (Table 2), decreasing RNA content is a target for improving P-use efficiency (Veneklaas et al., 2012). There is variation in the fraction of non-storage P allocated to RNA among species for P-replete organisms, suggesting at first sight that there is scope for decreasing the RNA content (Table 2) The variation in RNA as a percentage of total non-storage P in P-replete organisms ranges from 46 to 65%, assuming (legend to Table 2) that, when only data on total nucleic acids are provided, RNA is 80% of the total nucleic acids. The highest percentage RNA is found in the cyanobacterium *Synechococcus* with a very low percentage of phospholipids, related to the large fraction of thylakoids in the total membranes with galactolipids and sulfolipids rather than phospholipids. However, the values in Table 2 are not normalized to growth rate which is an important component of the Growth Rate Hypothesis (Sterner and Elser, 2002).

**Table 2:** **Percentage of total non-storage phosphorus in the major phosphorus-containing fractions of a cyanobacterium, a green microalga, an aquatic flowering plant and four terrestrial flowering plants as a function of phosphorus supply and organ age**.

**Organism**	**P status**	**% Ester P^#^**	**% Lipid P**	**% Nucleic Acid P**	**References**
*Synechococcus* sp. (Cyanobactria)	Replete	21	6	DNA 8, RNA 65	Grillo and Gibson, 1979
*Synechococcus* sp. (Cyanobactria)	Limited	17	5	DNA 15, RNA 63	Grillo and Gibson, 1979
*Ankistrodesmus braunii* (Chlorophyceae)	Replete	19	32	DNA 4,RNA 46	Kanai and Simonis, 1968
*Spirodela* sp. (aquatic flowering plant)	Replete	24	24	52^*^	Bieleski, 1968a
*Spirodela* sp. (aquatic flowering plant)	Limiting	16	32	52^*^	Bieleski, 1968b
*Hordeum vulgare* (terrestrial flowering plant, leaf)	Replete	19	19	62^*^	Chapin and Bieleski, 1982
*Hordeum vulgare* (terrestrial flowering plant, leaf)	Limiting	35	23	42^*^	Chapin and Bieleski, 1982
*Lotus pedunculatus* (terrestrial flowering plant, leaf)	Replete	15	32	53^*^	Hart and Jessop, 1983
*Lotus pedunculatus* (terrestrial flowering plant, leaf)	Limiting	15	28	57^*^	Hart and Jessop, 1983
*Trifolium repens* (terrestrial flowering plant, leaf)	Replete	18	29	53^*^	Hart and Jessop, 1983
*Trifolium repens* (terrestrial flowering plant, leaf)	Limiting	14	29	57^*^	Hart and Jessop, 1983
*Trifolium repens* (terrestrial flowering plant, young leaf)	Replete	13	22	65^*^	Hart and Jessop, 1983
*Trifolium repens* (terrestrial flowering plant, older leaf)	Replete	19	28	53^*^	Hart and Jessop, 1983

# *Ester P = low-molecular-mass water-soluble phosphate ester*.

* *Reference only cites total nucleic acid phosphorus; approximately 10% DNA, 90% RNA*.

*Modified from Table 4 of Raven (2012)*.

The data in Table 2 also show that P limitation has a variable effect among species on the fraction of non-storage P allocated to nucleic acids (predominantly RNA), so it is possible that there is genetic variation in how P allocation responds to P limitation. However, the available data are very limited. Nevertheless, it is worth examining the possibility of decreasing the quantity of P in RNA while retaining such desirable attributes in crops as growth rate and yield, radiation- and water-use efficiency and resistance to biophages, underlies subsequent sections of this paper. This, and other, possibilities for economizing on P, have been examined elsewhere (Veneklaas et al., 2012; Raven, 2013c). Here, the emphasis is on the possibilities of economizing on RNA, as might be expected from the title of this paper, while acknowledging that there are other possibilities such as decreasing the concentration of phospholipids, low-molecular-mass water-soluble phosphate esters and, perhaps especially, stored inorganic P (Veneklaas et al., 2012; Raven, 2013c).

Veneklaas et al. (2012; see also Raven, 2013c) suggested that, for any low-molecular-mass water-soluble phosphate ester present at a steady-state concentration insufficient to saturate the enzyme, which metabolizes it, the metabolic flux through the phosphate ester could only be maintained if the concentration of the phosphate ester was further decreased by increasing the concentration of the enzyme, which is not saturated with the ester. Veneklaas et al. (2012) argued that, with optimal allocation, the P saved from decreasing the concentration of phosphate esters would, at least in part, be offset by greater P allocation to the RNA needed to synthesize the additional enzyme.

Decreasing the concentration of membrane phospholipids is another possibility for economizing on P (Raven, 2012, 2013c; Veneklaas et al., 2012). The role of phospholipids in non-plastid membranes can be replaced to varying extent by other polar lipids, generally the galactolipids and sulfolipids found in plastid, and especially thylakoid, membranes. This occurs constitutively in some cyanobacteria and algae, and can also occur more widely by acclimation to P deficiency in embryophytes as well as algae and cyanobacteria (Veneklaas et al., 2012; Raven, 2013c). Since this acclimatory replacement of phospholipids by other polar lipids is reversed upon resupply of P, it is likely that the alternative polar lipids have some structural and/or functional traits, which are in some way inferior to phospholipids, although why the phospholipids are superior is not clear (Lambers et al., 2012; Veneklaas et al., 2012).

A final possibility for economizing on P is to decrease the concentration of stored inorganic P. There is clearly variation between cell types in the their role in P storage: P is stored in mesophyll cells but not epidermal cells in leaves of cereals such as *Hordeum vulgare*, and also in the proteaceous *Hakea* adapted to very low P availability (Leigh and Tomos, 1993; Karley et al., 2000; Shane et al., 2004). Veneklaas et al. (2012) indicate ways in which the level of stored P could be decreased. Such reduction is possible in the context of agriculture, where environmental P availability can, within limits, be assured. However, retention of the P (including stored P) in harvested products, be they forage or grain for domesticated animal or human food, is important. Furthermore, such decreases in stored P might not be appropriate in wild plants or algae living in environment with fluctuating P availability.

Before proceeding with consideration of optimal allocation and the Growth Rate Hypothesis it is important to consider the different means of expression of the RNA concentration of photosynthetic organisms (Table 3), and the maximum peptide elongation rate per ribosome. Optimal allocation and the Growth Rate Hypothesis are then considered in relation to growth rate as a function of RNA concentration, and temporal and spatial changes in net protein synthesis and protein turnover. The paper concludes with consideration of how protein synthesis rate, and hence growth rate, could be increased by maximizing protein synthesis rate per unit RNA at all times and in all parts of the organism.

**Table 3:** **References for RNA content of tissues of photosynthetic organisms on various bases**.

**Basis for RNA Content**	**Organisms**	**References**
RNA: DNA	*Scenedesmus*	Rhee, 1973
	Eukaryotic microalgae	Dortch et al., 1983
	Cyanobacteria	Bertilsson et al., 2003
	Eukaryotic microalgae	Nicklisch and Steinberg, 2009
	Mangroves	Reef et al., 2010
	Marine macroalgae	Reef et al., 2012
RNA: protein	*Pinus*	Matzek and Vitousek, 2009
	Eukaryotic microalgae	Nicklisch and Steinberg, 2009
RNA:biomass	*Scenedesmus*	Rhee, 1973
RNA: volume	*Scenedesmus*	Rhee, 1973
RNA P:organism P	*Spirodela*	Bieleski, 1968a,b
	*Ankistrodesmus*	Kanai and Simonis, 1968
	*Scenedesmus*	Rhee, 1973
	*Synechococcus*	Grillo and Gibson, 1979
	*Hordeum*	Chapin and Bieleski, 1982
	*Lotus*	Hart and Jessop, 1983
	*Trifolium*	Hart and Jessop, 1983
RNA:fresh weight	*Laminaria*	Mizuta et al., 2003
RNA:cell	*Microcystis*	Nagai et al., 2011

## Bases for the measurement of RNA content for comparison with growth rate

Many different bases have been used for measuring the RNA content of plants, including (eukaryotic) algae and cyanobacteria (Table 3). The most obvious way of relating RNA content to specific growth rate in the Growth Rate Hypothesis is via RNA per unit protein (e.g., Matzek and Vitousek, 2009). With measurements of specific growth rate, these measurements allow calculation of the mass of protein synthesized per unit time per unit mass of RNA. Converting this into amino acyl units added per ribosome per second involves further assumptions explained by Karpinets et al. (2006). Such measurements and calculations allow estimates of the maximum achieved rate of catalysis by ribosomes in organisms under resource-replete conditions, as discussed in the next Section. Such calculations require that the RNA:protein ratio is derived from measurements of RNA and of protein made in absolute units such as mass per cell or mass per unit biomass. Where RNA:protein is given in relative units (e.g., Nicklisch and Steinberg, 2009) it is not always clear whether the relative units for RNA bear the same relationship to mass of RNA as do the relative units for protein to the mass of protein, so it is best not to use such relative ratios to calculate number of amino acyl units added per ribosome per second.

RNA:DNA ratio (Table 3) has been used as an indicator of growth rate: more rapid growth means more rapid protein synthesis with attendant need (in an optimally allocating organism) for more rRNA, mRNA, and tRNA per unit DNA (e.g., Mizuta et al., 2003; Nicklisch and Steinberg, 2009; Reef et al., 2010, 2012) This is best applied intra-specifically, since comparison between even closely related species might be misleading as a result of, for example, large variations in genome size (Mbp), despite their all having a similar number of unsilenced genes. With a large genome, the increment in RNA:DNA for a given increment in specific growth rate is expected to be smaller than for a close relative with a smaller genome, granted a similar cell size. However, the positive correlation of genome size with cell size (non-vacuolar intracellular volume) (Hodgson et al., 2010) means that, for a given increment in specific growth rate, more RNA is needed per cell (and hence per unit DNA) in the larger cells. This can be related to the finding by Zhu et al. (2005) that there is a positive correlation between rRNA copy number in the genome and cell length among microalgal cells of a range of cell sizes, with a fraction of cell volume occupied by vacuoles in the largest cells. There is a slight inverse correlation between cell size and specific growth rate in microalgae (Figure 3 of Finkel et al., 2010), so in microalgae it seems that RNA:DNA should be constant for a given specific growth rate independently of genome (and cell) size. These arguments are related directly to unicellular organisms on a per cell basis, or to RNA per individual cells in colonies of varying size such as *Microcystis* (Table 3). For multicellular organisms, it is predicted that, for a given organism size the increased genome size is balanced by a decreased number of cells, so the RNA:DNA in the whole organism should be independent of genome size for a given specific growth rate. The same prediction should apply to unicellular organisms on a biomass basis. Another problem with interpreting the RNA:DNA ratio data is with very fast-growing bacteria. Here the generation time can be less than the time needed to replicate the genome from the single initiation point, so that there are two or more overlapping rounds of replication, so DNA per cell increases with implications for interpreting the RNA:DNA ratio.

A further basis for expressing the RNA concentration of organisms is biomass (Table 3), such as dry weight, fresh weight or volume (e.g., Mizuta et al., 2003). In the absence of estimates of protein concentration, measurements of the (specific) growth rates, measured as the rate of increase in the units used to reference RNA concentration, and with the rates manipulated by resource supply, could be used to test the Growth Rate Hypothesis.

In conclusion, the various methods for expressing RNA content (Table 2) all have applicability to relating growth rate to RNA, and hence RNA-P, concentration, provided the growth rate is expressed in the units used as the basis for measurements of RNA, and the various caveats mentioned above are borne in mind.

## The maximum peptide elongation rate per ribosome achieved *in vivo*

The relevance of the maximum rate of peptide elongation per ribosome (PER) to the Growth Rate Hypothesis and optimal allocation of P is that a higher (specific) growth rate requires a higher rate of protein synthesis, and hence either a higher specific reaction rate of polypeptide elongation (amino-acyl units added per ribosome per unit time) or more ribosomes per unit biomass. The maximum specific reaction rate is apparently set by a trade-off between the rate of peptide-chain elongation and the accuracy of translation (Ehrenberg and Kurand, 1984; Kurland, 1987; Koch, 1988; Xia, 1996). The ribosomal process of pairing of amino-acyl-charged tRNA and mRNA is not the only cause of error in translation; errors in transcribing the DNA base sequence into the sequence of mRNA, and in charging tRNAs with their appropriate amino acids, are the other places in which errors can arise (Reynolds et al., 2010; Imashimizu et al., 2013; Yadavalli and Ibba, 2013). Too low an accuracy of translation with high specific reaction rates could involve the use of additional ribosomes to make replacement proteins, a fraction of which will also be non-functional, thus offsetting in part or whole the saving of rRNA as a result of their high specific reaction rate (Raven, 2012; see also the section on protein turnover). The trade-off between rate and accuracy are more generally modulated by elemental resource (other than P) availability (Xia, 1996; Bragg et al., 2012) and by the trade-off between reaction rate and energetic efficiency, with higher efficiency involving greater resource input to synthesize enzymes and other catalysts (Raven, 1984; Raven et al., 2013).

If the ribosomes are operating at their maximum specific reaction rate set by the trade-offs described above (see Karpinets et al., 2006), a positive correlation is predicted between the RNA:protein ratio and growth rate. The Growth Rate Hypothesis requires that, at low specific growth rates, there is an over-provision of protein relative to RNA, i.e., faster growth needs more protein synthesis machinery rather than more protein per unit biomass. While this is plausible for structural proteins, for catalytically-active proteins it implies that the specific reaction rate of these catalytic proteins is lower in slower-growing than in faster-growing cells. Decreasing the protein concentration of slower-growing cells of a given genotype, or cells adapted to very low-nutrient environments (oligotrophs, as opposed to copiotrophs: Koch, 2001), would require a more dilute solution of protein in the cytosol, as documented for bacteria from the oligotrophic ocean for comparison with copiotrophic bacteria (Button et al., 1998). The alternative for decreasing the protein per cell would be to decrease cell size, which is constrained by the genome size (Raven et al., 2013). Other aspects of genetically determined variations in growth rate among algae are considered by Flynn (2009). Further work is needed on the RNA concentration of genetically determined variations in growth rate among vascular plants (Lambers and Poorter, 1992), since there are variations in the RNA concentration in some metazoans in parallel with genetically-determined variations in growth rate (Elser et al., 2003).

The primary data from which *in vivo* PER (units of amino-acyl units added per ribosome per second) are the specific growth rate of the organisms and the protein and RNA content in g per organism. The g protein per organism multiplied by the specific growth rate gives the instantaneous rate of protein synthesis as g protein per organism per day; division by g RNA per organism gives g protein synthesized per day per g RNA. Conversion to units appropriate for calculating the PER requires correction of the total RNA content for the fraction which is rRNA (assumed to be 0.8 in the absence of specific measurements for the organism concerned: see discussion above), and the fraction of the ribosomes that are active (0.80 in eukaryotes, 0.80–0.90 in bacteria, assumed herein to be 0.080): Karpinets et al. (2006), Loladze and Elser (2011).

Table 1 of Karpinets et al. (2006; see also Loladze and Elser, 2011; Ehrenberg et al., 2013) summarizes the literature and lists specific growth rates and protein and RNA from per cell from which g protein synthesized per day per g RNA can be calculated. The *in vivo* rates are up to 88 g protein per g RNA per day (range of 0.59–21 amino acyl units added per ribosome per second t 37°C: Table 3 of Karpinets et al., 2006) for three data sets on different species of chemoorganotrophic Bacteria with their 70S ribosomes. Closely similar maximum rates are cited for *Escherichia coli* at 37°C by (Ehrenberg et al., 2013). For the eukaryotes (fungi), up to 35 g protein is produced per g RNA per day, with a range of 2.8–10 amino acids per ribosome per second (Karpinets et al., 2006) for (predominantly) 80S cytosolic ribosomes. The experimental organisms are three fungal studies involving species of chemoorganotrophic (non-lichenized) Ascomycota at 30°C. The range of values for peptide elongation rate per ribosome in Karpinets et al. (2006; see also Loladze and Elser, 2011; Ehrenberg et al., 2013) relate not only to the different organisms and techniques, including growth temperatures, used, but also to large ranges in specific growth rate imposed by external factors such as resource availability. Since the organisms were grown at temperatures from 30 to 37°C, and this paper deals with photosynthetic organisms, the peptide elongation rates have been normalized, assuming a Q_10_ of 2, to a temperature (20°C) more relevant to the growth conditions for many photosynthetic organisms. The rates obtained in this way are up to 27 g protein per g RNA per day (range of 0.3–6.5 amino acids per ribosome per second) for Bacteria and up to 17.5 g protein per g RNA per day (range of 1.4–5.7 amino acids per ribosome per second) for the ascomycotan Fungi. The highest values are the most appropriate for this analysis, since the focus is on the maximum catalytic rates, which can be achieved by the two sorts of ribosomes. It should be noted that the Q_10_ value of 2 may be an under-estimate for *in vitro* protein synthesis by ribosomes from teleost muscle, albeit over a lower temperature range than that considered for microorganisms. However, work on *Escherichia coli* (Farewell and Neidhardt, 1998) gave a Q_10_ of 183 for *in vivo* protein synthesis over the range 28–37°C.

The conclusion from this work is that the maximum PER, adjusted to 20°C assuming a Q_10_ of 2, is 6.5 for Bacterua and 5.7 for Eukarya. These values, for 70S and (predominantly) 80S ribosomes, respectively, will not be regarded a significantly difference.

More relevant to work on photosynthetic organisms are the data compiled from Lourenço et al. (1998, 2002, 2004) in Table 2 of Flynn et al. (2010) for a cyanobacterium and nine phylogenetically diverse species of algae. The values obtained are as g protein synthesized per g RNA per day for *in vivo* protein synthesis. The cells were grown on a 12L:12D light-dark cycle with temperatures of 23°C in the light and 20°C in the dark. The PER for these organisms can be calculated as follows.

Bacteria have 4560 ribonucleotides per 70S ribosome (Loladze and Elser, 2011). The mean *M*_*r*_ of ribonucleotide is 340 g/mol (Loladze and Elser, 2011), so the *M*_*r*_ is 4560 × 340 or 1550400 g per mol ribosomal RNA. Division by 6.10^23^ gives g per molecule: 1550400/6.10^23^ is 2.58.10^−18^, so 1/2.58.10^−18^,or 3.88.10^19^ ribosomes per g RNA (assuming all RNA is rRNA). The mean *M*_*r*_ of aminoacyl units in protein is 110 g/mol (Loladze and Elser, 2011), so there are 110/6.10^23^ or 5.45.10^21^ molecules per g protein. Applying this to *Synechococcus*, 62.5 g protein synthesized per g RNA per day, the g protein synthesis per second per g RNA is 7.23.10^−4^. This corresponds to a PER of 7.23.10^−4^. 5.45.10^21^/3.88.10^19^ or 10.19 amino-acyl units added per second per ribosome. This calculation assumes that all cellular RNA is rRNA. Since rRNR may only be 0.8 of total RNA (discussed above), and only about 0.8 of ribosomes are active (Karpinets et al., 2006; Loladze and Elser, 2011), a corrected value is 15,9 amino-acyl units added per second per active ribosome.

Making a similar calculation for the green alga *Tetraselmis*, the difference from the cyanobacterium is the greater RNA per ribosome. This means that there is 2332400 g per mol ribosomal RNA in eukaryotes (Loladze and Elser, 2011), or 1.5 time the value for bacteria. With 55.7 g protein per day per g RNA or 6.45.10^−4^ g protein produced per second per g RNA, the PER is 13.6 amino-acyl units per second per ribosome. Correction for the fraction of RNA which is rRNA, and the fraction of active ribosomes gives 21,25 amino-acyl units per second per ribosome. The PER is higher than for *Synechococcus*, although the cyanobacterium has a slightly high rate of protein synthesis per unit RNA per day than the green alga; the higher PER for *Tetraselmis* results from the higher RNA content of 80S ribosomes than of 70S ribosomes.

These values are significantly higher than the highest values cited by Karpinets et al. (2006) for chemoorganotrophic Bacteria and Fungi; the values would still be higher than for the chemoorganotrophs if there had been adjustment for the temperature in the light phase being just above 20°C. The rates of protein synthesis in the light and the dark phase of the diel cycle of photosynthetic organisms are discussed below. Since the significant number of data sets for PERs of chemoorganotrophs, and especially for Bacteria, are taken as the benchmark maximum values for PER (Karpinets et al., 2006; Loladze and Elser, 2011; Ehrenberg et al., 2013), the highest PERs for photolithotrophs constitute significant outliers. The reason(s) for this difference between the very high maximum values for photolithotrophs than for chemoorganotrophs need further investigation. An obvious possibility is greater fractional extractability of protein than of RNA from the photolithotrophs than the chemoorganotrophs. This seems more plausible than there being real differences between the specific reaction rates of 70S and of 80S ribosomes in chemoorganotrophs relative to photolithotrophs. The basis of any such differential extractability is not obvious. In eukarytes a possibility is that the differential extractability resides in the plastids which can contain a significant fraction of the cellular RNA and protein (discussed below). However, there is no obvious mechanism for such differential extractability. Furthermore, this suggestion as to the differential extractability of protein and RNA apparently cannot apply to cyanobacteria where all the ribosomes are in one compartment. However, this single compartment (the cytosol) is equivalent to the plastid stroma, since the plastids are evolutionarily derivatives from cyanobacteria, so that a mechanism applicable to plastid stroma could also apply to the cytosol of cyanobacteria. However, it must be emphasized that there is currently no mechanism for such differential extractability.

Differential extraction is not a problem with the *in vitro* protein synthesis experiments of Storch et al. (2005) where values of up to 34 g protein synthesized per g RNA per day in teleost muscle at 15°C were found. Assuming a Q_10_ of 2 (less than that reported by Storch et al., 2005 between 0 and 15°C), the rate at 20°C would be 48 g protein per g RNA per day which is still rather lower than the higher value found for the microalgae cited above, i.e., 62.5 g protein synthesized per g RNA per day. Such investigation s are important in relation to the finding (Raven, 2013c) that photolithotrophic growth has a low P-use efficiency (rate of biomass gain per unit P in an organism) than does chemoorganotrophic growth, This difference is seen in comparing chemoorganophc and photolithotrophic (growing photolithotrophically in the case of facultatively chemoorganotrophic organisms) members of the Bacteria and of the algae and protists (Raven, 2013c). For terrestrial vascular plants, the comparison is between the aboveground and belowground parts of photosynthetically growing flowering plants (Raven, 2013c). These outcomes for P-use efficiency are the reverse of the prediction for the RNA component of organism P based on the analysis of the maximum rate of protein synthesis per unit RNA for photolithotrophs and for chemoorganotrophs, although as discussed above this difference could relate to greater difficulty in extracting RNA relative to protein in photolithotrophs than in chemoorganotrophs.

In conclusion, further work is clearly needed to elucidate any differences for the maximum specific reaction rate of ribosomes on a P basis between 80S and 70S ribosomes, and between photolithotrophic and chemoorganotrophic growth and, more generally, between photolithotrophs and chemoorganotrophs.

## Relation of RNA concentration to growth rate

The requirement for faster protein synthesis per unit biomass for faster-growing organisms means, with optimal allocation as indicated by the Growth Rate Hypothesis, an increase in RNA:protein, RNA:DNA and RNA:biomass. Such correlations have been widely found for chemoorganotrophic organisms (Sterner and Elser, 2002; Elser et al., 2003), but there is more variably for photosynthetic organisms (Sterner and Elser, 2002; Elser et al., 2003; Matzek and Vitousek, 2009; Flynn et al., 2010; Reef et al., 2010, 2012; Loladze and Elser, 2011). Particularly important in the context of RNA and P is the response of organisms adapted to low and to high P availabilities and, for any organism, the effects on RNA concentration when the growth rate is restricted by limited P availability relative to that of other potentially growth rate-determining factors.

Flynn et al. (2010, their Table 1) analyzed the published data on RNA content per cell or per biomass for cultures of cyanobacteria and eukaryotic microalgae exposed to varied supplies of the resources light, inorganic carbon, combined nitrogen, phosphorus and, through variations in temperature, thermal energy. Ignoring the two values for obligately chemoorganotrophic algae, six entries showed clear agreement with the growth rate hypothesis, four showed less clear agreement, five showed clear disagreement, and one showed less clear disagreement. The only case in which P limited the growth rate did not show clear support for the Growth Rate Hypothesis, since growth rate is a saturating function of the RNA concentration (Rhee, 1973; Flynn et al., 2010). There is no clear relationship of the nature of other limiting factors to whether there is agreement with the Growth Rate Hypothesis: this is well-illustrated for light supply, for which there are the greatest number of data sets (Flynn et al., 2010).

Published too late to be included in the analysis of Flynn et al. (2010), Nicklisch and Steinberg (2009) examined the RNA, DNA, and protein concentration of five species of eukaryotic microalgae grown at a range of growth rates imposed by varying the supply of light and of P for all five, of combined N for three, and of Si for the only diatom investigated. All five showed a significant positive correlation of RNA:DNA with growth rate; four showed a significant positive correlation of RNA:protein with growth rate, and one showed a weak positive correlation of RNA;protein with growth rate. These data support the Growth Rate Hypothesis, regardless of whether P was the resource, which was limiting growth. Nagai et al. (2011) investigated natural variations in growth rate, and RNA per cell, of populations of the freshwater colonial cyanobacterium *Microcystis* over two growing seasons. The RNA content increased with growth rate, although it is not possible to relate individual data points to particular genotypes, nor can the trends be related to particular combinations of environmental factors. With these significant provisos, the data support the Growth Rate Hypothesis.

Turning to macroalgae, Mizuta et al. (2003; see also Bartsch et al., 2008) used a population of sporophytes of the kelp *Laminaria japonica* (Laminariales: Phaeophyceae) on ropes in coastal waters. They examined the seasonal variation in external P, blade elongation rate (corrected for erosion) and the P, RNA and DNA concentration of the basal, meristematic, part of the blade between late February (sea temperature 2°C) and early September (sea temperature 19°C). The external inorganic plus organic P concentration varied from 2.8 μ M in early March to 0.4–0.6 μ M in April–September. There is a general correlation of RNA concentration with total intracellular phosphorus concentration. However, the elongation rate in early June, when RNA concentration is at its lowest, is five times the minimum value, which is in September when the RNA concentration is 2.5 times the minimum value in June. There is the complicating factor of the greater than 15°C temperature increase between the time of the maximum elongation rate in February and the minimum elongation rate in September; also, there is a 3° temperature increase between June (minimum RNA concentration) and September (minimum growth). After normalizing the elongation rate data to 20°C assuming a Q_10_ of 2, these data do not support the Growth Rate Hypothesis. Any correlation of growth rate and RNA concentration with external P availability is complicated by buffering by internal P stores, and the large change in temperature is a significant complication. Furthermore, it is not certain that P, rather than N, limits growth in the summer.

Reef et al. (2012) measured the RNA per unit fresh weight, RNA:DNA ratio and relative growth rate in three species of macroalgae cultured for a week in unenriched seawater, and with addition of combined nitrogen or of P, after collection from the oligotrophic Great Barrier Reef. Nutrient addition significantly increased the RNA concentration of all three algae, but did not increase the relative growth rate or the RNA:DNA ratio. Data for one of the species supplied with a range of concentrations of P between the values for control and the fertilized treatments showed that growth could be enhanced at an intermediate concentration, but no nucleic acid concentrations were measured. These data do not obviously support the Growth Rate Hypothesis; further experiments on growth rate and nucleic acid concentrations at intermediate nutrient, and especially P, concentrations are needed.

Turning to vascular plants, Matzek and Vitousek (2009) studied *Pinus contorta* and *Pinus muricata*, and found that nutrient-replete plants had three times the RNA:protein ratio in their needle biomass than did low-nutrient plants, but that the plant relative growth rate for the nutrient-replete plants was less than twice that of the low-nutrient plants. Lower protein:RNA values were also found for two other treatments, which resulted in faster growth, but with no data given for relative growth rate. Matzek and Vitousek (2009) also compared 14 *Pinus* species with wide variations in their growth rates, and found no correlation of growth rate with RNA:protein. Reef et al. (2010) investigated the effect of fertilizer treatment vs. control on the leaf and vascular cambium RNA:DNA ratios and shoot linear extension rate of two species of mangrove tree. There was a positive correlation between linear extension rate of the shoot and the RNA:DNA ratio (Reef et al., 2010), but as a test of the Growth Rate Hypothesis it would have been preferable to express growth on the same biomass basis as the RNA content.

The analyses above show that there is not always a positive correlation between specific (=relative) growth rate and RNA per unit biomass (or RNA:protein or RNA:DNA) in photosynthetic organisms, although in a number of cases there are reasons why such a correlation might not occur, e.g., the work of Mizuta et al. (2003) and Reef et al. (2012). Relatively few of the data sets involve organisms whose growth rate is determined by P supply. In the cases where there were measurements of the effects of variations of P relative to that of other resources on the relation of RNA concentration to growth rate, it is found that there is no significant difference in the effects of P and other limiting resources.

A final point is that there is a “subsistence quota” of RNA, i.e., a finite RNA concentration in organisms, which are prevented from growing by resource limitation (e.g., Figure 3 of Waldron and LaCroute, 1975). This RNA is presumably used in protein turnover in the absence of net protein synthesis, as well as allowing renewed growth when the availability of the limiting resource is increased. This concept, though not usually under the name “subsistence quota,” also applies to organs such as mature leaves, which do not grow and have no net protein synthesis. Despite net CO_2_ assimilation in photosynthesis minus 24 h respiration, andnet import of soil-derived nutrients through the xylem, over a 24 h diel cycle, the elemental content is kept constant from day to day under constant environmental conditions by export of photosynthate and the derivatives of soil-derived nutrients (except much of the calcium and balancing anions) in the phloem to growing and storage zones. The RNA in mature leaves is involved in protein turnover, including synthesis to replace photodamaged components of photosystem II (Raven, 2011, 2012).

In summary, the increment of specific growth rate for unit increase in RNA concentration range from no increase in growth with increased RNA to one instance of a growth rate increment greater than the increment in RNA concentration; however, this latter finding is based on two data points and the quantitative effect is rightly not emphasized by the authors (Matzek and Vitousek, 2009).

## Temporal and spatial changes in the rate of net protein synthesis

The discussion here focusses on diel changes in microalgae, although there is also mention of longer-term ontogenetic and seasonal changes in multicellular organisms. For an optimally allocating photosynthetically growing organism under P limitation, it is predicted that that protein synthesis continues throughout the light-dark cycle. Such a continuous use of ribosomes would minimize the allocation of P to ribosomes per unit biomass for a given growth rate and protein concentration per unit biomass. Such a continuous functioning of ribosomes would not be in contradiction to a greater delivery of combined N to the growing shoot of terrestrial vascular plants in the light, when there is a higher transpiration rate, than in the dark, since there is the potential for storage of the temporary excess of combined N in the vacuoles of mature leaves, which are the main transpiring structures. There is a continuous movement of organic N in the phloem from mature leaves to growing zones. There is the possibility of an energetic penalty of this continuous protein synthesis, rather than being limited to the photoperiod, for greening tissues (Raven, 1985; Andrews et al., 2009).

For P-replete microalgae cultures in a diel light-dark cycle there is a large range in the fraction of total net protein synthesis which occurs in the photoperiod (reviewed by Raven, 1976a,b,c, 1984). In some algae, protein synthesis ceases in the dark, while in others it continues at essentially the same rate in the dark phase as in the light. The latter strategy would seem more appropriate for P limitation, since it allows use of ribosomes at the same rate as in the dark, taking into account the doubling of ribosome content for each cell doubling. Cuhel et al. (1984) measured protein synthesis rate, as ^35^S incorporation from supplied ^35^SO^2−^_4_, and showed that the rate of protein synthesis in the dark (night) in P-replete cultures of the green microalga *Dunaliella* was almost as high as that in the light. Lancelot et al. (1986) examined natural populations, and P-replete laboratory cultures with 12 h light: 12 h dark cycles, of the prymnesiophycean *Phaeocystis pouchetti*. This work showed that the rate of protein synthesis in the dark phase was equal to that in the light in cultures grown at light saturation, with a linear decrease in the dark:light ratio of the rates of protein synthesis with decreasing photosynthetically activity in the previous photoperiod until zero protein synthesis occurred in the dark despite a finite preceding photosynthetically active radiation.

Smith et al. (1990) showed that natural populations of Arctic sea ice algae synthesized protein in the dark phase at no more than 30% of the rate in the light period. More recent data are for nitrate and total particulate N in cells of P-replete marine diatoms (*Thalassiosira pseudonana, Thalassiosira rotula*, and *Thalassiosira weissflogii*) and the marine prymnesiophycean *Emiliania huxleyi* (Needoba and Harrison, 2004). The dark rates of particulate N production in the dark phase as a percentage of that in the light phase is 49% for *Thalassiosira pseudonana*, 31% for *Thalassiosira rotula*, 30% for *Thalassiosira weissflogii*, and 9% for *Emiliania huxleyi*. While these values cannot be directly related to protein-synthesis rates, the predominance of protein N in such cells (Lourenço et al., 1998) means that they are reasonable approximations of the protein synthesis rates. Again, it would be of interest to see if similarly low rates of protein synthesis in the dark are found in P-limited cultures. Although Berdal et al. (1994) measured growth rates, DNA, RNA, and protein concentrations of *Heterocasa* sp.in 12 h light: 12 h dark cycles in high-P and low-P cultures, no data are given for protein-synthesis rates in the light and dark phases. However, Berdal et al. (1994) showed that there was a higher protein synthesis rate per unit RNA in the low-P than in the high-P cultures in the exponential and post-exponential phases of batch culture. Needoba and Harrison (2004) point out that dark assimilation of nitrate by phytoplankton is consistent with diel (or longer period in larger, slower-growing cells) vertical migration by cells in habitats with little vertical water movements, thus presumably optimizing the acquisition of PAR near the surface in the photoperiod and nitrate (or, indeed, phosphate, or iron) near the thermocline (see Raven and Richardson, 1984; Raven, 2013a).

For terrestrial embryophytes the available data cover a longer time period than the diel period addressed for microalgae. This accords with the longer generation of the embryophytes than of the microalgae. For embryophytes there are data for DNA, RNA, with some studies of rRNA distinguishing between monosomes and polysomes and/or whether ribosomes are free or protein-bound, and studies of protein synthesis during leaf development (Loening and Ingle, 1967; Eilam et al., 1971; Lin and Stocking, 1978; Makrides and Goldthwaite, 1981; Dean and Leech, 1982). There are also measurements of the P concentration in apical tissues, leaves and stems (Moody and Edwards, 1978). Eilam et al. (1971) examined developing *Cucumis sativus* leaves, showing that the ribosome concentration peaked at about 14 days when the leaf had attained more than half of its final fresh weight. There was a predominance of larger cytosolic ribosomes early in leaf development, with an increasing fraction of the 70S (mainly plastidic) ribosomes, so that about 40% of the rRNA is in the 70S ribosomes. Similar change in cytosol and plastid had previously been found by Loening and Ingle (1967) in developing leaves of *Phaseolus vulgaris*. Leaves of this species were also used by Makrides and Goldthwaite (1981); they also found that organelle rRNA peaked 4.5 days after cytosol rRNA did, which is about the same time as the peak in total protein concentration, although the decline in total leaf protein concentration with age is less rapid than that for 80S or 70S ribosomes. Dean and Leech (1982) used *Triticum aestivum* leaves, and followed 70S and 80S ribosomes and (soluble) protein per cell as a function of mesophyll cell age. Total ribosomes per cell peaked before protein per cell, and 80S ribosomes peaked before 70S ribosomes.

There are relatively few seasonal studies on RNA in vascular plants, e.g., breakdown in senescing leaves and dormant tissues (Veneklaas et al., 2012). In leaves, the RNA produced in developing leaves and used there in net protein synthesis is often maintained at a higher level in mature and senescing leaves with no net protein synthesis than is needed to support protein turnover (Raven, 1989, 1994, 2011, 2012, 2013c; Lambers et al., 2010; Veneklaas et al., 2012).

Two conclusions can be drawn from these data about the extent to which ribosomes are used in protein synthesis. One is that the fraction of organelle ribosomes is greater than the fraction of the total proteome (by protein number) of the organism which is encoded by organelle genomes (Timmis et al., 2004; van Wijk and Baginsky, 2011). Even allowing for a number of highly-expressed genes in the organelles, e.g., Rubisco in (especially) C_3_ flowering plants (Raven, 2013b; Raven et al., 2013), this suggests that plastidic (and mitochondrial?) ribosomes are less active in protein synthesis than are cytosol ribosomes. The other conclusion is that total rRNA decreased after full expansion, despite the absence of net protein synthesis, presumably because RNA is needed for protein turnover. In addition to repair of photodamage to photosystem II (Raven, 2011), turnover of individual proteins occurs on a diel basis, as shown for young leaves of *Zea mays* by changes in the transcriptome (Jończyk et al., 2011) and the proteome (Feng et al., 2011).

All of this work on embryophytic plants involves P-replete conditions, and mainly those adapted to relatively high P supply. Experiments on these plants, and on algae, under P-limited conditions are needed. As for studies on algae, it would be of interest to use plants adapted to growth in low-P soils to see if there was more rapid developmental decrease in ribosome content, and the related possibility of a decreased diel turnover of proteins with a corresponding economy in ribosomes (Lambers et al., 2010). An excellent start was made by Sulpice et al. (2014) who studied species of the Proteaceae from Western Australian soils with very low P content and P availability and compared them with the ruderal *Arabidopsis thaliana* which requires higher P availability. The Proteaceae had much lower rRNA, and expecially, plastid rRNA, in developing leaves that did the Proteaceae. These Proteaceae have delayed greening, i.a. a considerable delay between leaf expansion and the development of photosynthetic competence, relative to *Arabidopsis* and many other plants from higher-P soils. Sulpice et al. (2014) argue that this allows recycling of P in rRNA from the ribosome population appropriate to leaf expansion into RNA in the ribosome population used in the development of photosynthetic competence. Such temporal recycling of RNA P presumably contributes, with the replacement of phospholipida by glycolpilids (Lambers et al., 2012) to the higher photosynthetic P use efficiency. (rate of photosynthetic carbon gain per unit leaf P) in the Proteaceae than in *Arabidopsis*.

Further work is needed to determine the genetic variation in the extent to which the maximum catalytic activity of ribosomes is used under P-replete and P-limiting conditions. This might allow breeding of organisms, which approach P economy in ribosome use of the most P-economical plants, at least for closely-related organisms. More general alteration in ribosome usage in increasing P-use efficiency could only come about with significant further research.

## Involvement of RNA, and hence of P, in protein turnover

The ribosome-use data discussed so far relates to net protein synthesis. However, there is significant protein turnover in all non-dormant cells, although all of the available data on photosynthetic organisms seem to relate to those that are P-replete. Turnover has been measured by tracer techniques on bulk protein; a review, with significant new data, is to be found in Quigg and Beardall (2003). There are also proteomics combined with tracer methodology on individual proteins (Martin et al., 2012; Mastrobuoni et al., 2012). These data are averaged over time; there are also data on the diel variations on a per cell or per unit biomass basis of the content of particular proteins. Diel variations in the content of enzymes of C and N (from combined N) metabolism in, for example, the diatom *Thalassiosira pseudonana* (Roberts et al., 2007; Brown et al., 2009; Granum et al., 2009; Ashworth et al., 2013). A further consideration is the synthesis and breakdown of cyclin proteins as the cell cycle proceeds; since *Thalassiosira pseudonana* in the work of Roberts et al. (2007) and Granum et al. (2009) were dividing more than once a day, the changes in the enzymes of C and N metabolism cannot be synchronized with that of the cyclin genes. In embryophytes there is also evidence of turnover of individual proteins occurring on a diel basis, shown for young leaves of *Zea mays* by changes in the transcriptome (Jończyk et al., 2011) and the proteome (Feng et al., 2011). As discussed above under consideration of the maximum specific reaction rate of ribosomes in polypeptide elongation, the replacement of non-functional and possible damaging proteins resulting from inaccurate translation, which could be increased by increased specific reaction rate of ribosomes, also requires additional ribosomes (Ehrenberg and Kurand, 1984; Kurland, 1987; Koch, 1988; Raven, 2012). The errors introduced at transcription are of the order of 1 in 10^5^ (Imashimizu et al., 2013), i.e. less than that of translation, which occurs at about 1 in 10^4^ (Reynolds et al., 2010; Yadavalli and Ibba, 2013), and replacement of these incorrectly synthesized proteins cannot accuount for the turnover rate of cellular proteins in microalgae which is about 0.03 of the rate of net protein synthesis (Quigg and Beardall, 2003).

The occurrence of protein turnover means that, assuming involvement of all ribosomes at their maximum peptide elongation rate, there is a greater need for rRNA per cell or per biomass than would be the case if there was no protein turnover. Such protein turnover also requires a greater content of mRNA, and presumably tRNA (Roberts et al., 2007; Brown et al., 2009; Granum et al., 2009; Ashworth et al., 2013) and tRNA. Protein turnover requires more mRNA, rRNA, and tRNA, and hence more P, on a cell or biomass basis.

Two further examples where there is a diel component of protein turnover concern the replacement of catalytically active proteins damaged during their functioning. The D_1_ protein involved in photosystem II photochemistry in all oxygenic can be photodamaged, especially when the photoprotective mechanisms cannot dissipate all of the excess excitation energy at high irradiances. The need for additional RNA for the synthesis of new protein to replace photodamaged proteins in photosystem II is considered by Raven (1989, 1994, 2011, 2012) and Lambers et al. (2010). The damage occurs in the light, although repair can extend into the dark phase (Raven, 2011). The involvement of RNA, and hence P, in the production of the photoprotective machinery is not necessarily related to the diel cycle (Raven, 2011).

A second example is nitrogenase, which can account for 5% of the total protein in diazotrophic cyanobacteria; it is an enzyme whose catalytically active form is inhibited by oxygen (Raven, 2012). Diazotrophic cyanobacteria show diel variations in nitrogen fixation. Heterocystous cyanobacteria, and the non-heterocystous marine *Trichodesmium*, fix nitrogen mainly in the photophase, while unicellular diazotrophic cyanobacteria such as the marine *Crocosphaera* confine their nitrogen fixation to the scotophase. Repair, and intrinsic diel synthesis and breakdown of nitrogenase, mean a diel variation in the synthesis of this protein (Raven, 2012). The involvement of RNA in the production of oxygen-protective mechanism, such as the heterocyst walls, does not necessarily vary with the time of day (Raven, 2012). The importance of the involvement of RNA, and hence P, in protein synthesis related to the oxygen sensitivity of nitrogenase, in cyanobacteria is underlined by the P co-limitation of cyanobacterial diazotrophy in some of the natural environments of diazotrophic cyanobacteria (e.g., Sañudo-Wilhelmy et al., 2001; Mills et al., 2004).

## Possibilities for economising on P use in ribosomes

As indicated above, P is regarded as an ultimate geochemically limiting resource for biological productivity, although in many places and at many times combined N, Fe, K, or S might be limiting for productivity. An underlying P limitation could, in evolution, result in optimal allocation of P among P pools in maximizing some outcome such as growth rate per unit P, i.e., the highest achievable P use efficiency, the capacity to compete with organisms at the same trophic level, and the ability to limit the attentions of biophages. As highly expressed genes, the ribosomal proteins can also show optimal allocation of elements involved in their DNA and mRNA (C, N) and the resulting proteins (C, N, S) (Bragg et al., 2012). There is, of course, no such possibility of economizing on P in ribosomes without extensive re-engineering of this catalytic structure.

Economizing on P use in RNA while maintaining growth rate and yield, radiation and water use efficiency and resistance to biophages, requires investigation, and remediation, of the following possible causes of “over-provision” of RNA.

1. Decrease the ribosome content of each cell compartment, or organ, to the minimum needed to accommodate the maximum likely rate of protein synthesis. It has been seen above that there are apparent cases of an overprovision of ribosomes in plastids, as well as the maintenance of higher ribosome concentrations in mature and senescent structures (with evidence from leaves) than are needed for protein turnover in maintenance processes [discussed above, and in Raven (2012) and Veneklaas et al. (2012)]. Cases of apparent over-privision of particular protein on a cell basis, e.g., of ribulose1,5-bisphosphate carboxylase-oxygenase, could also be addressed as a means of decreasing the required maximum rate of protein synthesis
2. Maintain protein synthesis at a constant rate throughout the diel cycle, as discussed above.
3. Minimize the extent of protein turnover, without incurring a corresponding increase in RNA use in providing, for example, additional photoprotection to decrease photodamage to PSII, or restriction of oxygen damage to nitrogenase.

## Conclusions and requirements for further research

The available data on oxygenic photosynthetic organisms lends significant support to the Growth Rate Hypothesis, although a substantial number of well-conducted investigations show no continuous increase in RNA concentration with increasing growth rate determined by the supply of external P or other environmental factors; further well-designed experiments are needed. In specific relation to limitation by P availability, the few available data sets do not give a clear picture on whether decreased RNA concentration is relatively greater than that of the other major non-storage pools of P, i.e., low-molecular-mass phosphate esters and phospholipids, and more focussed experimentation is needed. However, it would appear that in cases where the spatial and temporal allocation of RNA does not follow optimality, there could be economies in RNA and hence P. Examples are modification of the spatial allocation of RNA in relation to the required rate of protein, and of the timing of protein synthesis (including protein turnover) so as to give the same relative rate of protein synthesis during exponential growth, or the same rate of protein synthesis in linear growth; If achievable, these modifications would require less RNA per unit biomass to achieve the same growth rate; however, achieving such reallocations would not be simple. Furthermore, as with all such manipulations, there could be unintended consequences, so any resulting genotype should be examined under a very wide range of environment conditions.

### Conflict of interest statement

The author declares that the research was conducted in the absence of any commercial or financial relationships that could be construed as a potential conflict of interest.
